# Tapered Pillar Design for High‐Precision Force Readout in Miniaturized Engineered Heart Tissues From Human Pluripotent Stem Cells

**DOI:** 10.1002/adhm.202501664

**Published:** 2025-08-30

**Authors:** Milica Dostanić, Maury Wiendels, Laura M. Windt, Mervyn P.H. Mol, Francijna E. van den Hil, Richard P. Davis, Valeria Orlova, Berend J. van Meer, Massimo Mastrangeli, Christine L. Mummery

**Affiliations:** ^1^ Department of Anatomy and Embryology Leiden University Medical Center Leiden 2333 ZC The Netherlands; ^2^ The Novo Nordisk Foundation Center for Stem Cell Medicine reNEW Leiden University Medical Center Leiden 2333 ZC The Netherlands; ^3^ Department of Microelectronics Delft University of Technology Delft 2628 CD The Netherlands; ^4^ Sync Biosystems Leiden 2333 BD The Netherlands

**Keywords:** engineered heart tissues, human induced pluripotent stem cell‐based models, microfabrication, tapered pillars

## Abstract

Engineered heart tissues (EHTs) formed around flexible pillars are used to measure the contraction force of myocytes. When based on cardiac cells derived from human induced pluripotent stem cells (hiPSCs), EHTs capture human cardiac physiology and drug responses in vitro. However, variability in contractile function often arises due to variation in tissue positioning on the pillar. Here, novel tapered pillars are introduced to achieve spatial confinement of tissues in EHT devices. The devices are fabricated by moulding polydimethylsiloxane (PDMS) into micromachined tapered cavities of a silicon substrate. The symmetrically‐tapered geometry, with the minimum cross‐section at the pillar mid‐height, restricts tissue movement outside of the indented area. This increases sensitivity and accuracy of tissue contractile readout, providing high reproducibility with reduced variability between data points. Design and stiffness of tapered pillars are investigated to determine the optimal mechanical environment, obtain accurate contractile measurements, and achieve long‐term culture of EHTs. Results show that tapered pillars provide superior confinement efficiency (over 90%) compared to straight pillars (30%), with tissue confinement directly correlated to pillar geometry rather than stiffness. The optimized precision in force readouts and long‐term tissue studies enables higher sensitivity in the detection of contractile responses to drugs or diseases.

## Graphical abstract

Engineered heart tissues (EHTs) are a valuable approach in capturing human cardiac physiology and drug responses in vitro. Here, a novel tapered pillar design is developed in an EHT platform to confine tissues in a predefined position‐ at the middle of the pillar height. With tapered pillars, high sensitivity and accuracy of tissue contractile readout are achieved, and created a robust mechanical environment for EHT culture with reduced variability between individual data points.

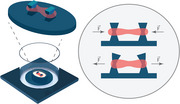

## 1 Introduction

EHTs are considered valuable in vitro models to study (patho)physiology of adult human cardiac tissue as they allow measurement of contraction force against a resistance, a functional readout of cardiac health.^[^ ^1^, ^2^, ^3^, ^4^ ^]^ When based on differentiated cells from patient‐derived hiPSCs, these in vitro microphysiological models often exhibit typical features of human cardiovascular disease, providing opportunities for research on drugs to treat these conditions.^[^ ^5^, ^6^, ^7^ ^]^ However, EHTs could benefit from design improvements that would increase efficiency and throughput while reducing costs.

Most EHT devices consist of two elastic anchoring points that facilitate the self‐assembly of muscle cell suspensions into tissue‐like bundles.^[^ ^8^, ^9^, ^10^, ^11^, ^12^ ^]^ The compacted tissues undergo spontaneous cyclic contractions and apply pressure to the elastic pillars, forcing them to bend. The pillar deformation can be converted into readouts of tissue contraction force and kinetics – hallmarks of cardiac tissue functionality. The main advantages of these models over other tissue culture modalities are: 1) high‐density multi‐cellular and multi‐cell type 3D microenvironment faithfully captures in vivo cardiac tissue complexity; 2) the passive load provided by the pillars guides tissue assembly and imposes cell alignment along the direction of tissue contraction; 3) the absolute force of contraction is an effective read‐out of such a system, that is not calculable from 3D in vitro cardiac models, such as cardiac micro‐tissues^[^ ^13^ ^]^ or cardioids.^[^ ^14^ ^]^

Though there are multiple examples of EHT devices^[^ ^9^, ^15^, ^16^, ^17^, ^18^ ^]^ to date no single, standardized EHT model or device design has been broadly adopted by academia, industry or regulators. Most research groups have developed and use their own custom‐made systems. These differ in size, number, and shape of pillars, tissue position, manufacturing method, material, cellular input, mechanical properties, among other factors. Some devices rely on the alignment of multiple parts, introducing additional assembly challenges and reducing reproducibility.^[^ ^19^, ^20^, ^21^, ^22^ ^]^ These technical issues are often a source of variability in experiments, which may compound with inherent tissue variability, leading to high dispersion of the contractile outputs unless multiple measurements (such as drug dose‐response curves) are made sequentially on the same EHT and masking detection of subtle physiological effects.^[^ ^23^, ^24^, ^25^ ^]^ We recognized an urgent need to de‐couple readout variability arising from technical limitations in device design and fabrication methods from the inherent readout variability of biological origin. We therefore sought to design and test a new and reproducible microenvironment for EHT culture in which experimental conditions are highly controlled.

The importance of fine‐tuning and precisely engineering the mechanical environment in the EHT platforms is even more apparent when considering chamber‐specific cardiac cell types in in vitro models. Depending on whether ventricular or atrial, cardiomyocytes might require different mechanical cues. For example, atrial tissue is subjected to lower tension, has lower work demands, and generates lower force of contraction than ventricular tissue which pumps blood around the body.^[^ ^9^, ^26^, ^27^ ^]^ Therefore, the ability to tune and adjust the mechanical properties of an EHT platform easily is an important advantage when developing such models.

**Figure 1:**
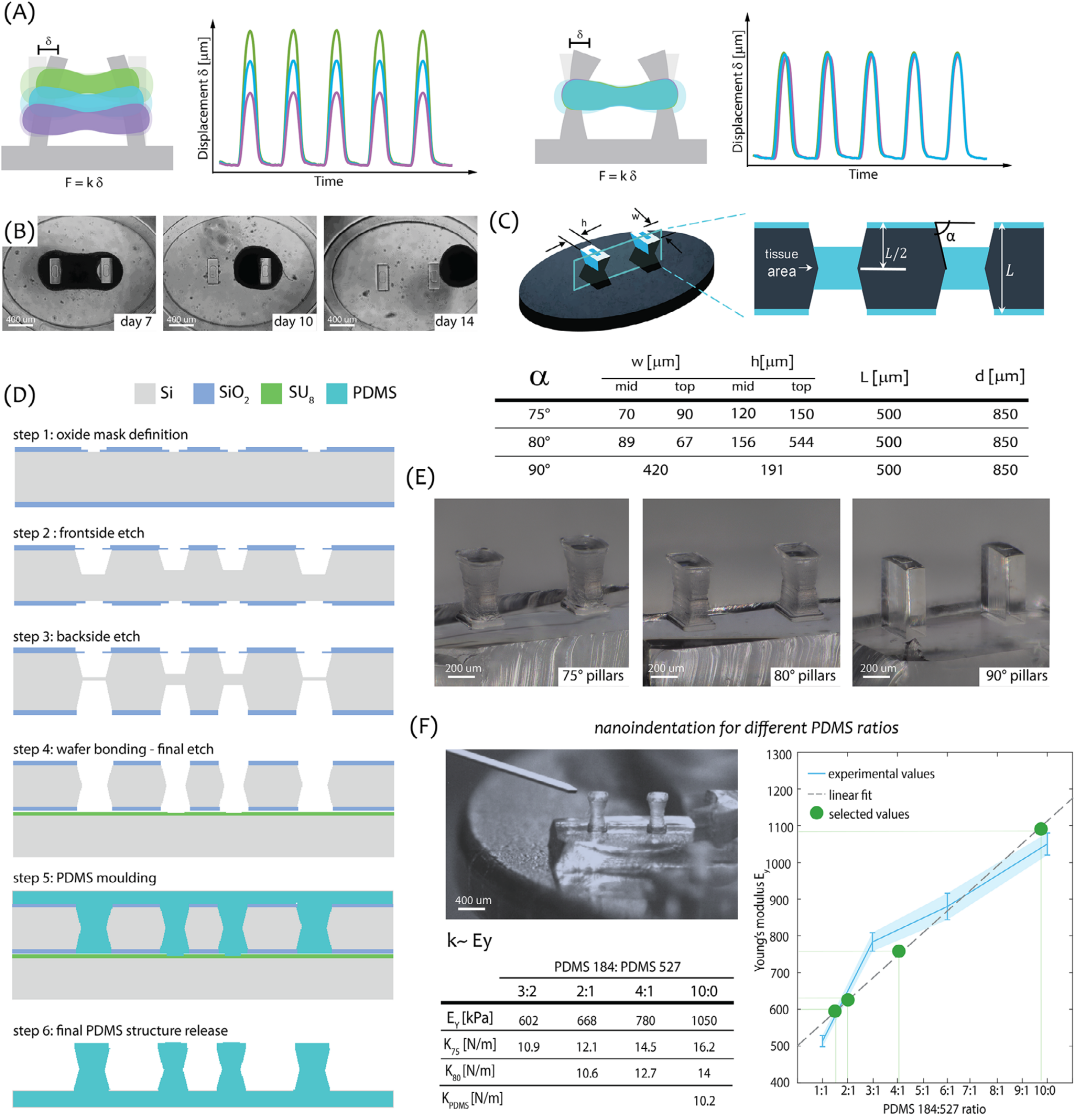
Fabrication and mechanical characterization of tapered pillars of varying stiffness: A) Illustration of contraction force relative to the tissue position along the pillar height in case of straight (left) and tapered (right) pillar geometry. B) Timeline of EHTs dislodging from the straight pillars. C) Design and geometrical parameters of three pillar designs (α = 75°, α = 80°, α = 90°). D) Cross‐sections of the microfabrication process of the PDMS‐based EHT platform with tapered pillars. E) Optical images of PDMS pillars of three different tapering angles α = {75°, 80°, 90°} released from the silicon mould; F) Mechanical characterization of tapered pillars fabricated with different PDMS 184:527 ratios using nanoindentation. The graph shows values of Ey obtained based on experimental data for different material ratios and the final material ratios extrapolated for Ey to determine the expected pillar stiffnesses. The final mechanical parameters are summarized in the table.

The starting point of this study was our downscaled version of the HeartDyno system,^[^ ^28^ ^]^ which we had previously demonstrated suitable for EHT formation.^[^ ^29^ ^]^ This device consists of two elastomeric pillars of uniform rectangular cross‐sections, positioned in the middle of an elliptic microwell.^[^ ^29^, ^30^ ^]^ The device was fabricated by combining standard cleanroom microfabrication techniques and moulding of PDMS. This enabled high reproducibility of the final structures and easy fabrication upscaling using wafer‐level manufacturing. Even though we demonstrated successful tissue formation, high cell viability, and measurable functionality in this device,^[^ ^30^ ^]^ we noted low overall reproducibility and yield in the experiments due to the EHTs moving upward, along the pillar height, resulting in progressive EHT displacement and eventual release from the pillars into the culture medium. The force data point was then lost from the assay. The outcome was also low accuracy in force measurement, as the pillar bending and, subsequently, the measurement of the absolute force of contraction are directly correlated with the position of the applied force, i.e., the position of the EHT along the z‐axis (Figure **1**A). Uncertainty in the EHT vertical position led to high variability of measured contractile parameters, low reproducibility, and low yield of data points from each experiment, as most of the tissues failed to reach the end of the two‐week experimental period. Prior devices reported tissue confinement by introducing special shapes on the tips of the pillars.^[^ ^21^, ^31^, ^32^ ^]^ However, most of them lack mechanical robustness, leading to low experimental yield, or have poor compatibility with optical tracking algorithms, which are sensitive to contrast between transparent pillars and opaque cardiac tissue.

To address these issues and improve the technical aspects of the tissue microenvironment, we proposed a novel pillar design that systematically promotes tissue localization and confines the tissues to a pre‐defined position along the pillar height^[^ ^33^ ^]^ By symmetrically tapering the pillar walls toward the middle of the pillar height, the pillar geometry mechanically restricts EHT movement outside of the indented region (Figure 1C). Preliminary studies supported the superior confinement provided by the tapered geometry.^[^ ^33^ ^]^ However, the precise origin and quantification of EHT confinement efficiency was unexplored and optimization of mechanical properties of the pillars was required.

Here, we present an in‐depth study of EHT formation and function on pillars of different geometries (Figure 1C,E), exposed to various mechanical conditions. The study investigates the origin and efficiency of tissue confinement in the tapered pillar design and optimizes the mechanical properties of pillars. The hypothesis was that this would result in a robust and reproducible experimental microenvironment, and consequently in high force measurement sensitivity and accuracy.

## 2 Results and Discussion

### 2.1 Tapered Pillar Mechanical Properties Fine‐Tuned Using Two‐Polymer Mixing

The new EHT devices with tapered pillars were fabricated by combining standard microfabrication techniques with polymer processing, as described previously.^[^ ^33^ ^]^ Briefly, the PDMS‐based EHT devices were cast in a 500 µm‐thick, 4‐inch silicon wafer mould with symmetrically‐tapered cavities. Tapering of the silicon cavities was achieved by alternating anisotropic and isotropic etching steps in a deep reactive ion etching process. The fabrication flowchart is shown in Figure 1D. After peel‐off from the mould, the fabricated EHT devices were bonded (one device per well) to the bottom of the wells of a 96‐well plate. Due to restrictions imposed by the microfabrication process, preferences for specific tapering angles, and preservation of tissue shape and cell/extracellular matrix (ECM) volume, the stiffness of the tapered pillars was higher than that of the original straight pillars made of the same PDMS formulation. The stiffness of tapered pillars was reduced by using a modified elastomer formulation to match the stiffness of the straight pillar design and enable direct comparison of the effect of geometry alone. The mechanical properties of the pillar were tuned by combining two materials of different stiffness: PDMS 527, with bulk Young's modulus *E_y_* ≈5 kPa, and PDMS 184 in a 10:1 elastomer‐to‐curing agent ratio with *E_y_* ≈1 MPa. The two materials, PDMS 184 and 527, were mixed in different ratios: 1:1, 1:3, and 1:6, respectively. Mechanical characterization of each pillar variant was performed using nanoindentation measurements to extract effective pillar stiffness *k* (Figure 1F).^[^ ^29^ ^]^ The effective stiffness was calculated as the ratio of the force applied at the middle of the pillar height (*L*/2) and the displacement of the pillar's tip. Young's modulus of pillars with different material composition was calculated starting from the obtained stiffness *k* using analytical and numerical models, implemented to capture the complex mechanical behavior of tapered pillar geometry.^[^ ^34^ ^]^ The material mixing ratios were then extrapolated from the graph in Figure 1F, for specific *E_y_* of pillars corresponding to the stiffness range of *k* = {10,12,14,16}N m^−1^.

The starting stiffness values for 75° and 80° tapered pillars were 16 and 14 N m^−1^, respectively; 75° tapered pillars were therefore fabricated in the set of *k* = {10, 12, 14, 16} N m^−1^, while 80° tapered pillars were fabricated in the set of *k* = {10, 12, 14} N m^−1^. The final values for *E_y_* and *k* are shown in the Table of Figure 1F.

### 2.2 Comparison of Three Pillar Geometries

#### 2.2.1 EHT Formation is Unaffected by Pillar Geometry

**Figure 2:**
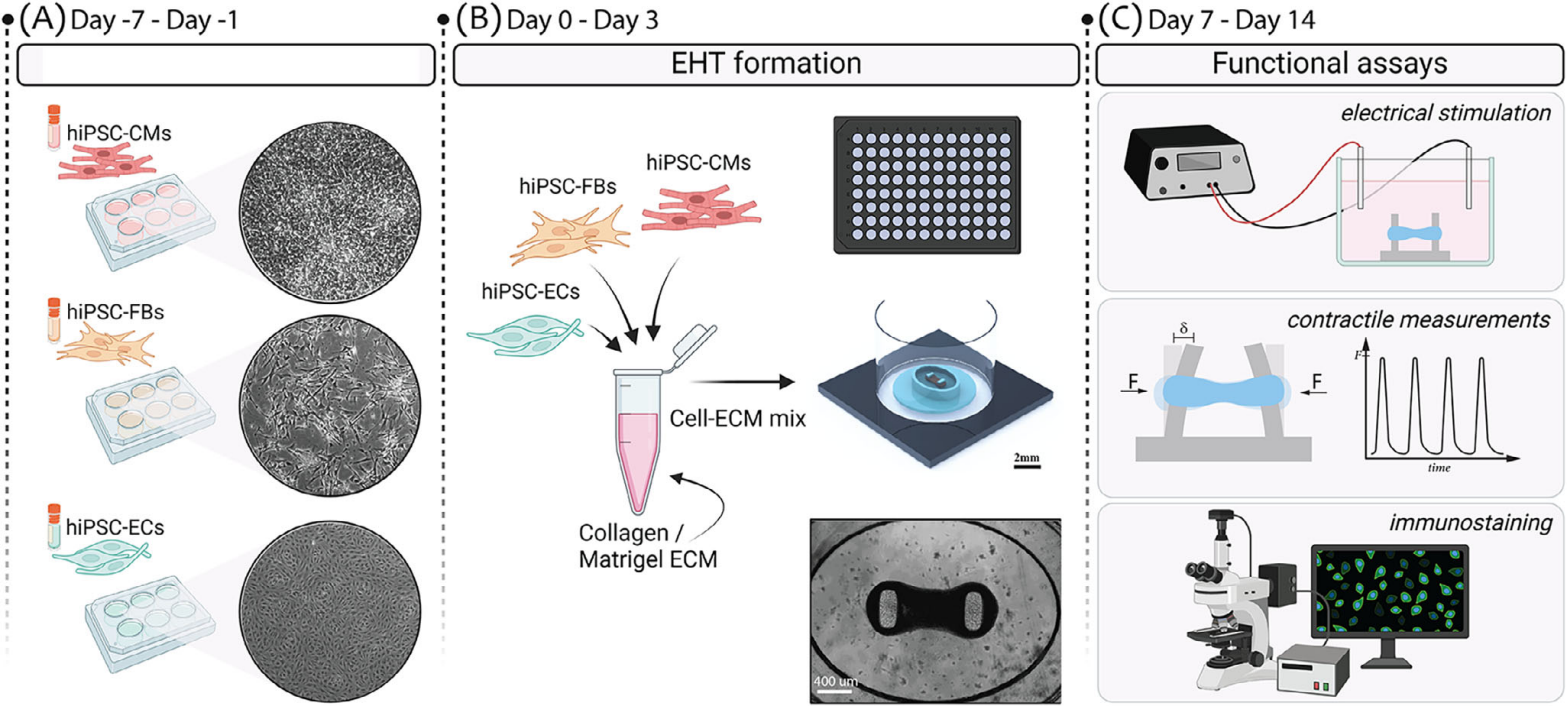
Graphical overview of the experiment timeline: A) Thawed hiPSC‐derived CMs, FBs, and ECs, on the day of tissue formation. B) Overview of EHT formation from hiPSC‐derived CMs, FBs, and ECs mixed within Collagen/Matrigel ECM (on the left) and seeded in 2 µL EHT device at the bottom of 96‐well plates (on the right). The EHTs form 72 h after seeding. C) Downstream assays to assess viability and functionality of EHTs over 14 days of tissue culture.

**Figure 3:**
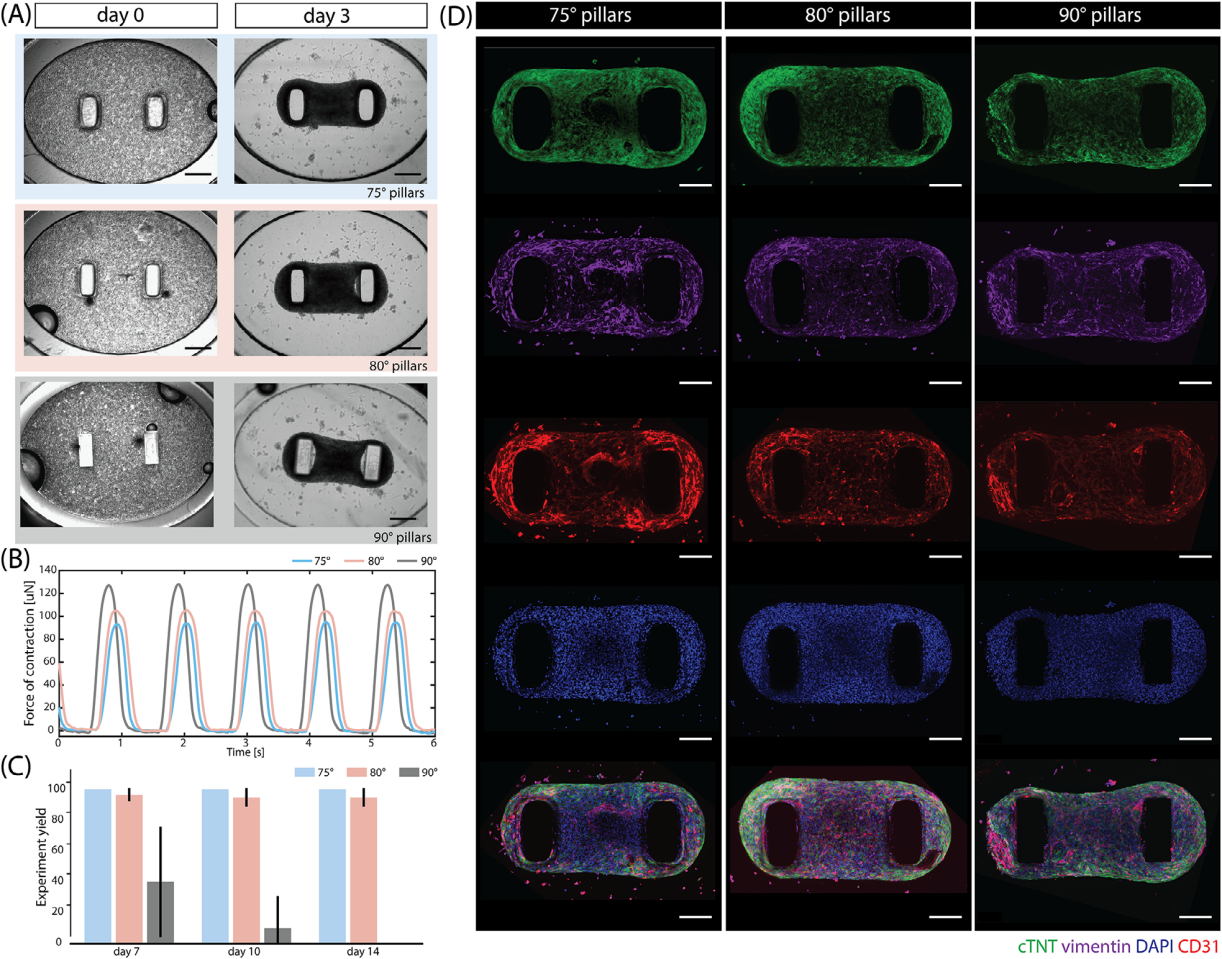
Tissue formation in different geometry pillars: (A) Phase contrast images of EHTs self‐assembly 1 h and 7 days after seeding the cell‐ECM mixture in the EHT devices with 75°, 80°, and 90° pillars. Scale bar: 400 µm. (B) Representative contraction traces of EHTs for the three different pillar designs (75°, 80°, and 90°), electrically stimulated at 1 Hz. (C) The experiment yield, i.e., percentage of tissues that remained on the pillars of different geometries throughout the experiment. (D) Representative immunofluorescent images of EHTs in devices with 75°, 80°, and 90° pillar designs stained for cardiac troponin T (cTNT) (green), vimentin (purple), CD31 (red), nuclei with DAPI (blue), and the merged image. Scale bar: 200 µm.

The formation and function of EHTs were quantified and compared among devices with three pillar geometries: straight (90°), 80° and 75° tapered pillars. The timeline of the experiment is shown in Figure **2**. The tapered pillar stiffness was tuned to match that of the straight pillars by mixing PDMS 184 and PDMS 527 in 2:1 and 3:2 ratios for 80° and 75° tapered pillars, respectively. The final effective stiffness of all three geometries was k = 10 N m^−1^. EHTs were formed by combining 70% hiPSC‐derived cardiomyocytes (hiPSC‐CMs), 15% hiPSC‐derived cardiac fibroblasts (hiPSC‐FBs), and 15% hiPSC‐derived endothelial cells (hiPSC‐ECs) in a Collagen/Matrigel–based ECM, resulting in 15.7 × 10^3^ cells per µL. The ratios of three cell types were adopted from the previous research from our group.^[^ ^13^, ^30^, ^35^ ^]^ Although the final ratios were established empirically to promote best cardiomyocyte maturation and microtissue stability, the initial rationale was based on the composition of the developing heart, where CMs are the dominant cell type (60–70%) and the rest of the (stromal) cells are mostly FBs and ECs. Each platform was seeded with 2 µL cell/ECM suspension containing 31.5 × 10^3^ cells per tissue. EHTs successfully formed in all three platforms (**Figure** **3**A), with the cell/ECM mixture self‐assembling into dense tissue‐like bundles around the pillars within the first 72 h, demonstrating that the change in pillar geometry did not affect tissue compaction around the pillars. The tissues remained functional for 14 days and their contractile kinetics could be measured throughout this period. Spontaneous contraction was evident from day 3 onward; however, based on our previous work,^[^ ^30^ ^]^ contractile assays were performed on days 7, 10, and 14 when tissue performance was optimal. Representative contraction traces are shown in Figure 3B.

Immunofluorescence staining for each cell type in the EHTs (cardiac troponin T (cTNT) for hiPSC‐CMs, vimentin for hiPSC‐FBs, and human CD31for hiPSC‐ECs) confirmed that there was no difference in cell distribution among platforms with different pillar geometries. Cell nuclei were visualized using DAPI (Figure 3D).

#### 2.2.2 Tapered Pillar Design Provides Superior Tissue Confinement

The efficiency in spatial tissue confinement of pillars was determined as the yield of experiments, quantified by counting the number of tissues that remained on pillars at different time points of the experiment. From the graph in Figure 3C, the experiment yield increased from ≈40% on day 7 for 90° pillars, to more than 97% for both tapered geometries. This difference was even more striking on day 10, with only a few tissues left on the 90° pillars (≈10%), while the yield for both tapered designs remained higher than 95%. Finally, by day 14, with no EHTs remaining on the 90° pillars (i.e., 0% yield), both tapered designs retained ≈95% of EHTs. Tapered pillars showed consistently high efficiencies in spatial confinement of EHTs in the middle of the pillar for the entire experiment duration. In this study, 75° pillars showed slightly higher confinement efficiency (100%) than those of 80° (≈95%), though this was not significant.

#### 2.2.3 Similar Contractile Parameters Between Three Pillar Geometries

**Figure 4:**
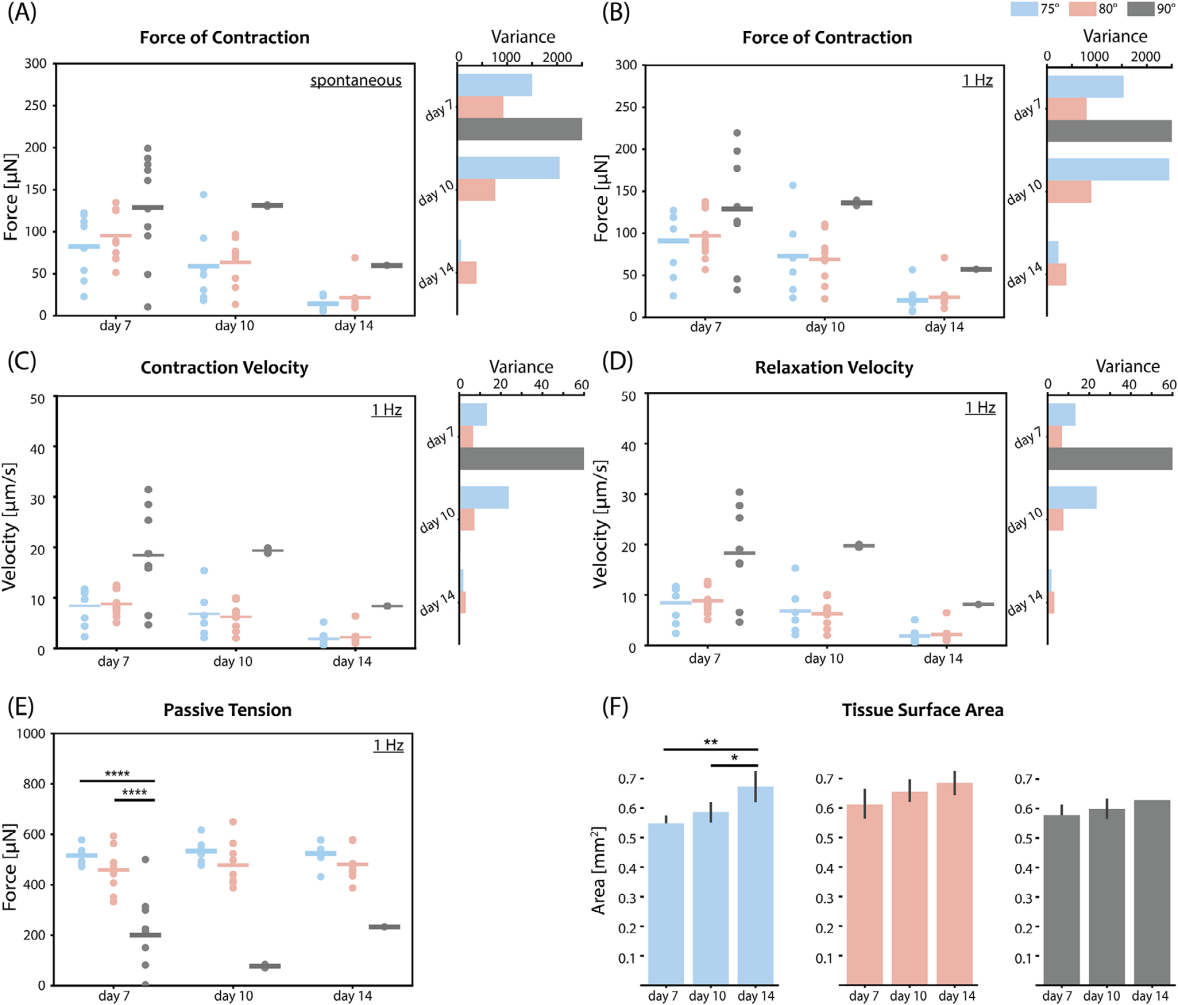
Comparison of contractile performance of EHTs on different geometry pillars (75°, 80°, and 90°) on days 7, 10, and 14: A) Spontaneous force of contraction and corresponding data variance of EHTs; B) Force of contraction and corresponding data variance for EHTs electrically stimulated at 1 Hz. Speed of contraction C) and relaxation D) with corresponding data variance calculations for EHTs electrically stimulated with 1 Hz. E) Passive tension generated by EHTs on different geometry pillars, electrically stimulated at 1 Hz. F) Comparison of tissue surface area for EHTs on different geometry pillars. N = 3; (*p < 0.05, **p < 0.005, ***p < 0.0005, ****p < 0.00005. Data is shown as mean ± SEM. N indicates EHTs from 3 independent batches per group, including *n* = 3 tissues per batch. One‐way ANOVA with Tukey post hoc test is shown.

Tissue contractile performance was then analyzed in more detail. Videos of contracting tissues were recorded for all three geometries on days 7, 10, and 14, under spontaneous contraction and electrically‐paced conditions. The contraction parameters were obtained using ForceTracker^[^ ^36^ ^]^ analysis by tracking the pillar tip displacement in the case of 90° pillars, and the mid‐height displacement for tapered geometries. By precisely focusing mid‐height (i.e., exactly at the location of applied load by tissue), the unique advantage of tapered geometry in improving the force measurement accuracy was evident (**Figure** **1A**). Moreover, the tapered geometry eliminated the “V‐neck” effect present in 90° pillars (Figure 3A), increasing the speed and quality of the contraction video analysis. The measurements of tissue contractile properties are summarized in **Figure** **4**. Figure 4A,B compares the absolute force of contraction of EHTs in pillars of different geometries, in spontaneously beating and paced regimes. The average force values showed no statistically significant difference among the data sets; however, the variance of the contraction force values was considerably reduced for both tapered geometries, likely due to the mechanical confinement of the EHTs. As a result, the force of contraction could be calculated more accurately for tapered geometries than straight pillars. The high variability of the data points in the case of 90° pillars reflects this inaccuracy in calculation arising from irreproducible tissue positioning. This is evident in the passive tension of the EHTs (Figure 4E). The large data spread indicates the varying load EHTs experience on straight pillars, while the force calculation assumes the tissue to always be in the middle of the pillar. The reduction in variability of the passive load on tapered geometries is also evident in this graph, again indicating the benefit of spatial confinement.

The same reasoning can be applied when analyzing the contractile kinetics measurements, shown in Figure 4C,D. The large spread in contraction and relaxation velocities for 90° pillars stems from the different starting points of EHTs along the pillar height. For example, tissues closer to the tip will cause much larger pillar displacement during a single contraction cycle, compared to the tissues positioned lower on the pillars, for the same force of contraction, as they experience lower resistance. The distance that pillars travel during a contraction cycle is directly translated into the speed of contraction and relaxation, explaining the high average values and large spread of measured velocities in the case of 90° pillars. Data points on EHT contractile parameters on days 10 and 14 are lacking as the tissues were not retained on the pillars, as expected from the experiment yield graphs in Figure 3C. Both tapered geometries, by contrast, showed stable and low‐variability measurements of contraction kinetics.

Finally, the change in tissue area was estimated and shown in Figure 4F. Although in all three pillar geometries there appeared to be a slight increase in the surface area, the difference was only statistically significant between timepoints in the case of 75° tapered pillars. It was possibly due to cell proliferation during the experimental analysis period.

### 2.3 Comparison of Tapered Pillars with Different Load

#### 2.3.1 EHT Formation Successful Under Different Load Conditions

The next study aimed to analyze tissue functionality under various passive loads and investigate optimal mechanical properties for the tapered geometries. EHTs were formed on 75° and 80° tapered pillars which had been fabricated in a range of stiffnesses. In the case of 75° pillars, four pillar types were fabricated with stiffness respectively of 10, 12, 14, and 16 N m^−1^. For 80° geometry, the stiffnesses were 10, 12, and 14 N m^−1^, resulting in three different pillar variants. The upper limit for the stiffness was determined by the stiffness of the pillars fabricated in PDMS 184. As previously, EHTs were formed by mixing the hiPSC‐CMs, ‐ECs, and ‐FBs in a 70:15:15 ratio, within Collagen/Matrigel‐based ECM. Within 72 h, the cell/gel mixture had successfully compacted into tissue bundles around the pillars, demonstrating that the difference in passive load did not negatively influence EHT formation.

#### 2.3.2 Contraction Force Variability Minimized for High‐Stiffness Pillars

**Figure 5:**
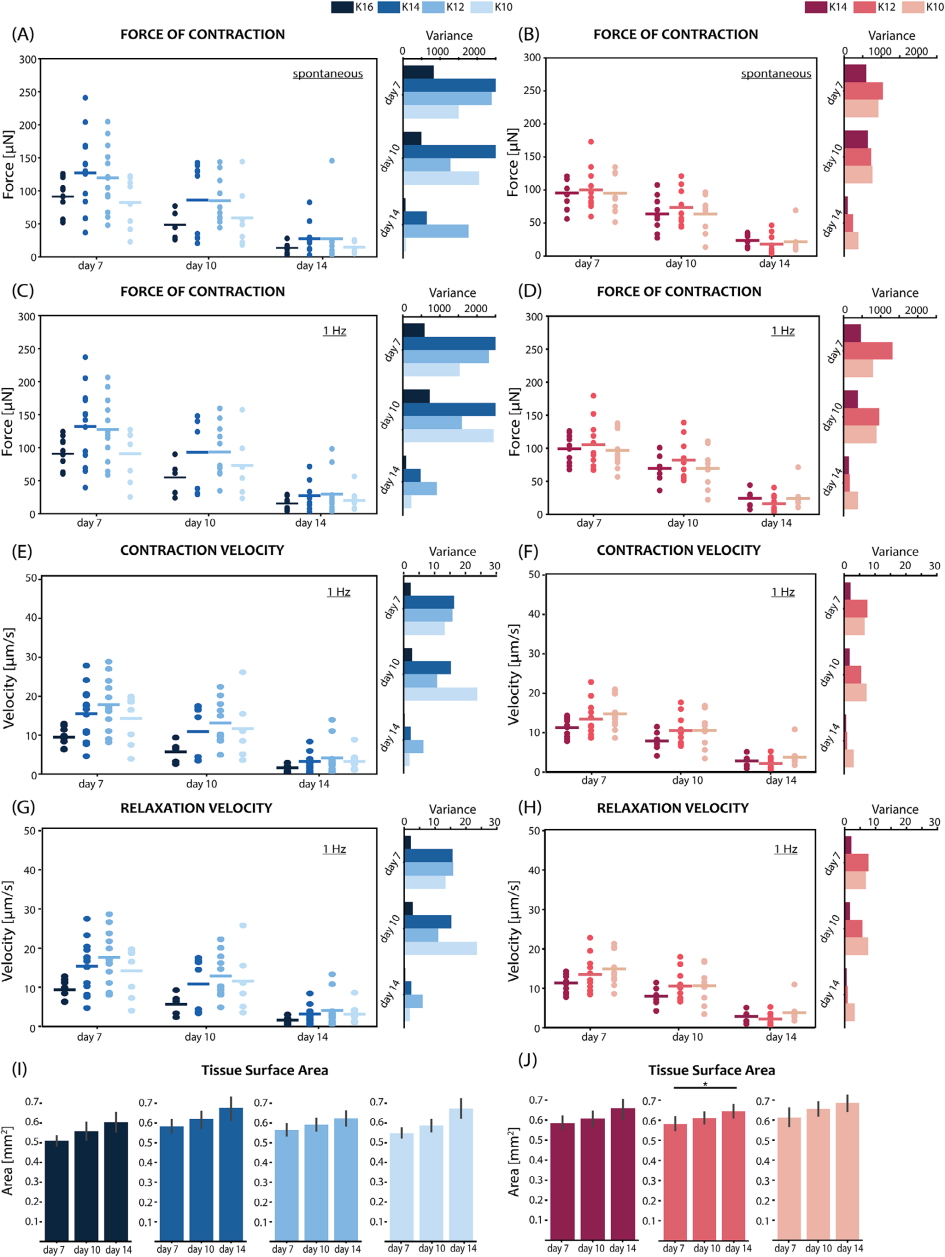
Comparison of contractile performance of EHTs on 75° and 80° tapered pillars of different stiffness on days 7, 10, and 14: Spontaneous force of contraction and corresponding data variance of EHTs on A) 75° and B) 80° tapered pillars. Force of contraction and corresponding data variance for EHTs electrically stimulated with 1 Hz pacing on C) 75° and D) 80° tapered pillars; Speed of contraction with corresponding data variance for EHTs electrically stimulated with 1 Hz pacing on E) 75° and F) 80° tapered pillars. Speed of relaxation with corresponding data variance for EHTs electrically stimulated with 1 Hz pacing on G) 75° and H) 80° tapered pillars. Comparison of tissue surface area for EHTs on I) 75° and J) 80° tapered pillars. N = 3; (*p < 0.05, **p < 0.005, ***p < 0.0005, ****p < 0.00005. Data is shown as mean ± SEM. N indicates EHTs from 3 independent batches per group, including *n* = 3 tissues per batch. One‐way ANOVA with Tukey post hoc test is shown.

As in the previous study, the contractile assays on the EHTs were performed on days 7, 10, and 14, under spontaneously beating and paced conditions. The comparison of absolute force of contraction among 75° tapered pillars, providing four different passive loads, is shown in **Figure** **5**A,C. Even though there was no statistically significant difference in the average force values among the conditions, the variance analysis in Figure 5A,C shows the largest decrease in the data spread for the highest‐load pillars (16 N m^−1^), compared to the other three types. The difference remained significant during the experiment, reaching the maximum value on day 7.

The complementary analysis was performed for 80° geometry and the corresponding force of contraction comparison is shown in Figure 5B,D. Again, similar average force values were observed between the conditions and data variance decreased significantly with the increase in pillar stiffness. The minimum variance values were reached for the highest stiffness pillars (*k* = 14 N m^−1^). The reduction in variance was slightly lower than in the case of 75° pillars; however, the trend remained the same, confirming that the lowest measurement variability is achieved for the highest stiffness pillars. The difference in variance became even more apparent and consistent during the experiment with the electrical pacing. The lowest variance was noted on day 7.

The analysis of contractile kinetics of the EHTs under the paced and spontaneously beating conditions on different load pillars is shown in Figure 5E–H. The contraction and relaxation speed graphs follow the trend shown in force graphs, demonstrating the same effect of passive load on tissue performance. The variance in the case of the highest stiffness 75° tapered pillars (*k* = 16 N m^−1^) was reduced by 80% compared with three other cases (*k* = 14,12,10 N m^−1^). Similar conclusions can be drawn for 80° tapered pillars. There was no significant difference between the average values of contractile kinetics parameters; however, a difference in variance was evident. The highest‐stiffness pillars show the lowest variability of values among the three types. The decrease in variance for the highest stiffness 80° pillars is more than 70% of the values in the other two conditions (*k* = 12,10 N m^−1^). The reduction of measurement variability in contraction and relaxation speed was even more prominent than that on contraction force, for both tapered geometries, which could be important for revealing subtle drug effects or disease phenotypes directly correlated with tissue kinetics.

Lastly, the change in tissue area over time was quantified and is shown in Figure 5I,J. Although the tissue size appeared to increase, the difference was not statistically significant for the 75° geometry. For 80° tapered pillars, the difference was significant though only for *k* = 12 N m^−1^ between day 7 and day 14.

### 2.4 Optimal Mechanical Environment for EHT Culture

#### 2.4.1 Higher Load Pillars Increase the Passive Tension in EHTs

Finally, the passive tension and corresponding contractile work generated in tissues was analyzed under different loading conditions. These two parameters showed the strongest distinctions between the conditions.

**Figure 6:**
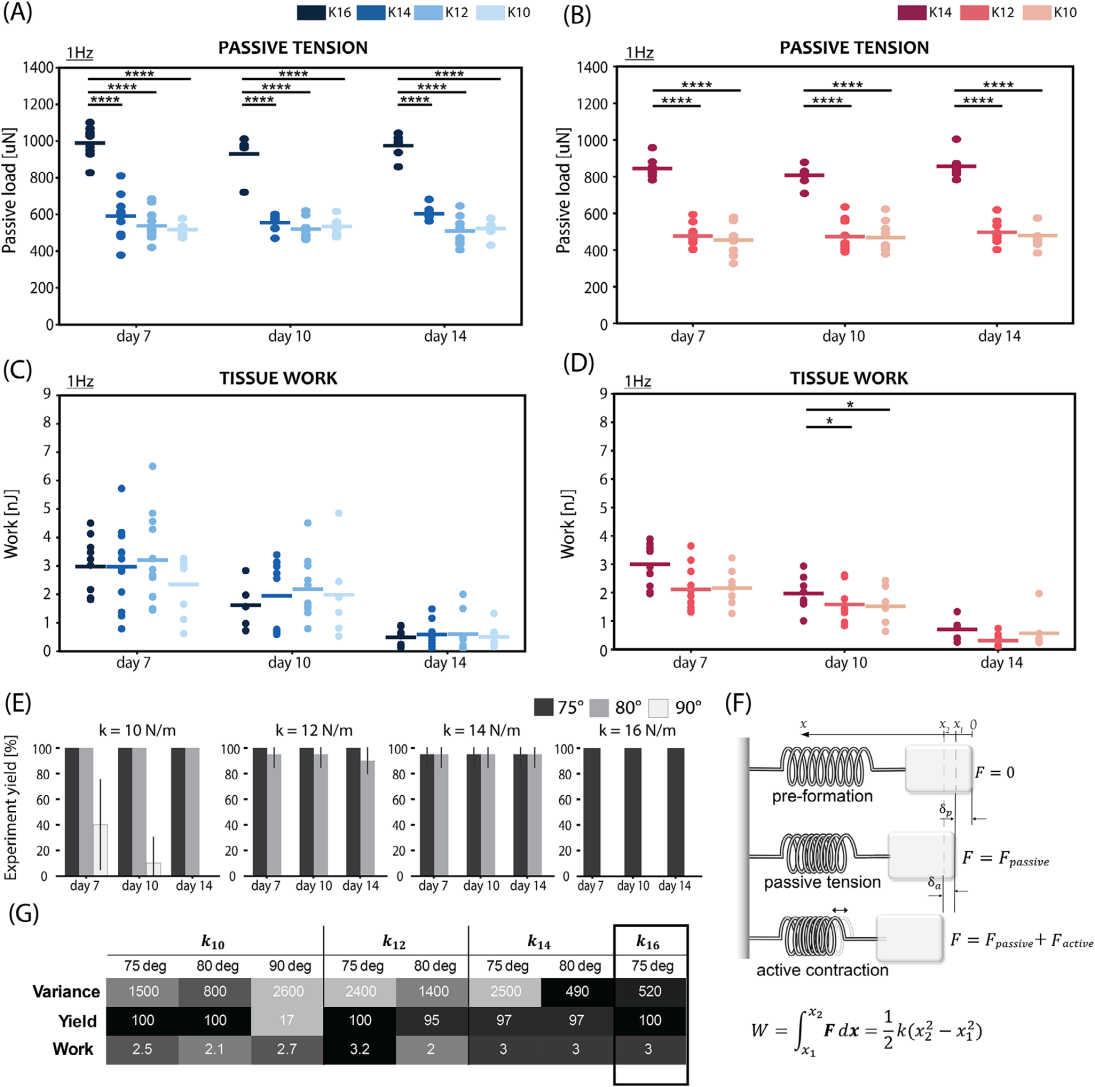
Passive tension generated by EHTs on A) 75° and B) 80° tapered pillars of different stiffness, electrically stimulated at 1 Hz. Work generated by EHTs on C) 75° and D) 80° tapered pillars of different stiffness, electrically stimulated at 1 Hz. N = 3; (*p < 0.05, **p < 0.005, ***p < 0.0005, ****p < 0.00005. Data is shown as mean ± SEM. N indicates EHTs from 3 independent batches per group, including *n* = 3 tissues per batch. One‐way ANOVA with Tukey post hoc test is shown. E) Comparison of the experiment yield for different pillar stiffness and geometry. F) Illustration of the simplified tissue mechanics model used for approximate work calculations. G) Summary of the tissue performance on 75° and 80° tapered pillars of different stiffness.

Passive tension generated in EHTs is the initial compaction force that tissues generate to counteract the opposing load imposed by the pillar stiffness. This tension is measured during the resting period of the contraction cycle, as the difference in the inter‐pillar distance with and without formed tissue. **Figure** **6**A,B show the passive tension comparison between different stiffness pillars for both 75° and 80° tapered geometries. The passive tension is significantly increased when EHTs are subjected to the high load condition: 16 N m^−1^ for 75° and 14 N m^−1^ for 80° tapered pillars. The remaining conditions showed no significant differences. The passive tension of EHTs was higher for *k* = 16 N m^−1^ in 75° tapered pillars than for 14 N m^−1^ in 80° pillars, as expected.

#### 2.4.2 Tissues Respond with Higher Work to Higher Load Demands

The work generated by tissues under different loading conditions was calculated using the pre‐loaded spring model from the theory of solid mechanics.^[^ ^37^, ^38^, ^39^, ^40^, ^41^, ^42^, ^43^ ^]^ The graphic illustration of the model is shown in Figure 6F. The model is a simplified representation of dynamic tissue behavior, assuming only the elastic nature of the tissue and neglecting the effect of the viscous part. Such an assumption was justified by the significantly diminished viscous response of tissue during active contraction.^[^ ^43^ ^]^ The approximation enabled inclusion of both passive and active force components into the generated work. Figure 6C,D show the work performed by the tissues under different loading conditions, in both tapered geometries. In the case of the 75° tapered pillars, the total work graph appeared to be a linear superposition of the active and passive force. No significant difference was found between the four loading conditions, demonstrating that tissues generate similar work in all pillar stiffnesses. Figure 6D shows a notable difference for 80° tapered pillars between the work output under the highest loading condition (*k* = 14 N m^−1^), compared to the other two conditions. This difference is apparent only on day 10.

#### 2.4.3 75° Highest Stiffness Tapered Pillars are the Optimal Design for the EHT Device

Lastly, the yield of experiments on different load pillars and geometries was quantified and shown in Figure 6E to determine the origin of EHT confinement efficiency and its relationship with pillar stiffness. This showed that successful confinement of EHTs on the tapered pillars is a direct consequence of the tapered geometry and not of the pillar stiffness. The experiment yield was high (>85%) for all tapered geometry conditions, regardless of the stiffness. Overall, 75° tapered pillars showed a slightly higher experiment yield than the 80° ones. In all 75° variants, except for *k* = 14 N m^−1^, the yield was 100%. In the case of the 80° pillars, a maximum yield of 100% was found for the softest pillars (*k* = 10 N m^−1^).

Comparison of the different pillar geometries and stiffness is summarized in the Table in Figure 6G. The tissue performance was compared and graded based on: variance of contraction force, yield of the experiment, and generated work. The measurement variance defines the sensitivity of the contractile readouts and the tissue confinement efficiency. The yield of the experiment measures robustness of the system and determines the duration and statistical power of the experiment. Finally, the work is used as the guiding parameter to find the optimal mechanical microenvironment that maximizes the tissue contractile output. The pillar ranking is shown in Figure 6G, concluding that the 75° tapered pillar with the highest stiffness (16 N m^−1^) is the highest‐scoring pillar type based on the study and grading criteria.

## 3 Conclusion

In this study, we proposed a new type of pillar for EHTs and optimized their mechanical properties in our miniaturized EHT device by addressing their geometry and material composition. Mechanical optimization was performed within the constraints imposed by the fabrication method and physiological affinity. The design we developed systematically imposed precise tissue positioning through tapered pillar geometry, increased the data point yield, and created a robust and reproducible microenvironment for EHT culture with minimized readout variability. The tapered geometry restricted the tissue movement outside the indented area to the middle of the pillar.

First, we showed that the tissue formation and function were not negatively affected by 80° and 75° wall inclinations versus standard straight pillars. Both tapered designs were superior in tissue spatial confinement, compared to the straight pillar platform. EHTs were stable on the tapered pillars for 14 days but not on straight pillars. This control of tissue position greatly improved accuracy in contraction force measurements. Furthermore, tapered geometries reduced variability in contractile values measured and increased measurement sensitivity, meaning that subtle differences in contractile parameters could, in principle, be revealed. In addition, the “V‐neck” effect reported earlier (Figure 3A)^[^ ^36^ ^]^ for straight pillars is eliminated in case of tapered geometry. The effect arises because EHTs do not adhere fully to the inner side of the pillar with a transparent shadow appearing next to the pillar interfering with downstream image processing. Correction for this effect normally requires manual processing, slowing down the quality and speed of the contraction video analysis, which has been solved using tapered geometry.

A further technical advance was fabricating a range of mechanical conditions in tapered pillar geometry, so that the tissues could be subjected to different passive loads. Mechanical load tuning was performed by combining PDMS variants.

Culturing EHTs on different stiffness pillars clearly demonstrated that the main contribution to the tissue confinement efficiency comes from the pillar geometry itself, rather than its stiffness. The study further revealed that the highest‐stiffness pillars yielded the lowest variability in contractile output and consequently the highest measurement sensitivity. As expected, passive tension generated by tissues increased significantly as stiffness increased. Regardless, the tissues maintained similar contractile output and adjusted to the higher load demands. In the case of 80° tapered pillars, the tissues even increased the generated work when subjected to 14 N m^−1^ passive load, compared to the less stiff conditions. Finally, considering all the relevant parameters, we concluded that the 75° tapered pillar geometry of 16 N m^−1^ stiffness was optimal for the EHT platform, regarding both the geometry and mechanical properties of the pillars. This design also yielded much higher experimental efficiency than conventional straight pillars.

Overall, the study showed distinct benefits for EHT device design that are relatively straightforward to implement at low overall cost and technical expertise for a microfabrication laboratory or, once moulds have been produced, for on‐site production in a standard cell biology facility.

## 4 Experimental Section

### Tapered Pillar Fabrication

Tapered pillars were fabricated using a combination of microfabrication and silicon processing, as previously described.^[^ ^33^ ^]^ Briefly, the tapered symmetrical cavities were etched into a 4‐inch diameter, 500 µm‐thick Si wafer by alternating isotropic and anisotropic deep reactive ion etching (DRIE). The tapering angle was precisely controlled by tuning the duration of each etching step to obtain a staircase‐like profile. The process started by defining a hard mask for DRIE in a SiO_2_ layer, obtained by plasma‐enhanced chemical vapor deposition (PECVD). The 5 µm‐thick oxide layer was deposited on both sides of the double‐side polished Si wafer. The oxide was then patterned using standard photolithography steps to create a two‐step etching mask for the tapered cavities (Figure 1D, step 1). The wafer was symmetrically etched from both sides, to achieve the minimum cross‐section area in the middle of the pillar height (Figure 1D, steps 2,3). The etching was finalized by bonding the wafer to a carrier using a 5µm‐thick layer of SU8 (Figure 1D, step 4). The final EHT devices with tapered pillars were obtained by casting PDMS formulations into the hydrophobic Si mould coated with a self‐assembled monolayer of perfluorosilane deposited from vapor phase (Figure 1D, steps 5,6). The stiffness of tapered pillars was tuned by mixing two elastomers of different mechanical properties: PDMS 527 and PDMS 184. The basic PDMS 184 was obtained by mixing the monomer with its curing agent in a 10:1 ratio. The expected *E_y_* for PDMS 184 in this case was estimated to be ≈1 GPa, based on previous measurements.^[^ ^29^ ^]^ The basic PDMS 527 was obtained by mixing its two components (A and B) in a 1:1 ratio. The expected *E_y_* of this material was estimated at 5 kPa based on literature.^[^ ^44^ ^]^ The two materials were then mixed in certain ratios (3:2, 2:1, 4:1, 10:0) to obtain the tapered pillar stiffness in the required range of *k* = {10,12,14,16}N m^−1^.

### Characterization of Pillar Mechanical Properties

A FemtoTools Nanomechanical Testing System (FT‐NMT03) was used for the mechanical characterization of tapered pillars made of different PDMS types, as described in the previous work.^[^ ^29^ ^]^ Three ratios of PDMS 184 and PDMS 527 mixtures (1:1, 3:1, 6:1, 10:0) were initially selected to cast tapered pillars into the Si moulds. Controlled force was applied along each pillar height, at different locations, and the pillar displacement was measured with a piezo mechanical sensor integrated into the tip of the nanoindenter probe. (**Figure** **2**F) The effective stiffness of pillars was obtained from the force‐displacement curve. Due to the complex geometry of tapered pillars, numerical and analytical models have been implemented to correlate the measured effective stiffness of pillar *k* **to the Young's modulus (*E*_*y*_) of its material. The analytical model was based on variable stiffness members^[^ ^45^ ^]^ and its complete derivation was described in ref. [34]. The numerical model was implemented using the Finite Element Method in COMSOL Multiphysics. These models resulted in the calculation of *E*_*y*_ for the range of expected pillar stiffness (*k *= {10,12,14,16} N m^−1^), for both tapered geometries. Finally, the PDMS 184:527 ratios were extrapolated from the graph in Figure 2E, based on the numerically obtained *E*_*y*_ values corresponding to each stiffness in the range.

### Generation of EHTs

CMs, cardiac FBs, and ECs were differentiated into respective cell types using the same hiPSC line (LUMC0020iCTRL‐06), according to the previous protocols.^[^ ^13^, ^35^, ^46^ ^]^ The purified cell populations were characterized using flow cytometry, and only the batches with >90% purity were used in experiments. (Figure S2, Supporting Information) The hiPSC‐CMs were cryopreserved on day 14 after the start of the differentiation and stored at −180 °C until the tissue generation. On day −7 before EHT generation, the hiPSC‐CMs were thawed on growth factor‐reduced Matrigel (75 mg mL^−1^, Corning) coated 12 well‐plates (≈2 × 10^6^ per 4 cm^2^) and cultured in mBEL medium. The hiPSC‐FBs were cryopreserved at −180 °C after the third passage from the begining of differentiation. On day −5 before EHT generation, the hiPSC‐FBs were thawed on vitronectin‐coated six‐well plates (≈2 × 10^5^ per 10 cm^2^) and cultured in Fibroblast Growth Medium 3 (FGM3, PromoCell). The hiPSC‐ECs, cryopreserved at −180 °C on day 9 of differentiation, were also thawed on day −5 before EHT generation on a gelatine‐coated six‐well plate (≈1 × 10^5^ per 10 cm^2^) and cultured in Endothelial Cell Complete Growth Medium (EC‐CGM). By day 0, all the cell types had recovered from thawing, with hiPSC‐CMs spontaneously contracting, and wells containing the hiPSC‐FBs and ‐ECs being confluent. The cells were dissociated according to previously described protocols,^[^ ^35^ ^]^ counted, and mixed in a final ratio of 70% hiPSC‐CMs, 15% hiPSC‐ECs, and 15% hiPSC‐FBs. After centrifuging at 1100 rpm for 3 min, the cell mixture was resuspended in formation medium^[^ ^15^ ^]^ and added to an ECM gel mixture containing pre‐mixed acid‐solubilized collagen I (41%, 3.3 mg mL^−1^, Sigma Aldrich), 6% NaOH, 5% DMEM (10x) and 9% Matrigel (10 mg mL^−1^, Corning). The gel composition and cell‐gel mixture ratios were taken from the protocol developed by.^[^ ^28^ ^]^ The final cell density in the cell‐gel mixture was 15.7 × 10^3^ cells µL^−1^. The EHTs were generated from 2 µL of cell per gel mixture, resulting in a total of 31.5 × 10^3^ cells per tissue. The EHTs compacted within 72 h and started spontaneous rhythmical contractions. For the first 3 days, tissues were cultured in formation medium. On day 3 the medium was switched to mBEL medium supplemented with endothelial cell and fibroblast growth factors, VEGF (50 ng mL^−1^) and FGF (5 ng mL^−1^), respectively. This medium was refreshed every 72 h.

### Contraction Assays

Video recordings of contracting EHTs were captured on days 7, 10, and 14 following EHT formation, using the Nikon Eclipse Ti inverted microscope connected to a high‐speed Thorlabs USB 3.0 digital camera. Videos were recorded for 10 s with a frame rate of 100 fps, focusing on the middle of the pillar height for tapered geometries, since that was the smallest cross‐section area. In the case of the straight pillars, the recording focus was only on the tip of the pillars, since the lack of features along the uniformly straight walls prevent the user from doing otherwise. It was argued that the superior focusing accuracy provided by the tapered pillars was an additional aspect of the higher measurements accuracy they enable. The difference in pillar displacement measurement position and effective stiffness for straight and tapered geometries was considered during force calculations. EHT contraction recordings were obtained in a custom‐built environmental chamber at 37 °C and 5% CO_2_. The videos of spontaneous tissue contractions and their response to the electrical stimulations were recorded. The pacing setup consisted of a pair of external platinum electrodes, which were immersed into a single well of a 96‐well plate. Electric field tissue stimulation was performed by applying bipolar rectangular pulses of 20 V peak‐to‐peak amplitude, 10 ms pulse duration, and 1 and 2 Hz pulse repetition rate. The tissue culture medium was refreshed after each pacing experiment. The force of contraction and contractile kinetics parameters were calculated using the custom‐made software ForceTracker.^[^ ^36^ ^]^ The software detects and automatically tracks the displacement of the pillars upon tissue contraction throughout the frames of recorded videos. The measured displacement was then correlated to the tissue force of contraction, considering the device‐specific parameters. Tissue contraction parameters of interest are provided in an output spreadsheet file for further graphical representation.

### Immunofluorescence Staining

For the immunofluorescence staining, the EHTs on pillars were washed with PBS and fixed for 1 h in 4% paraformaldehyde (PFA) on a rocking platform, at room temperature. The PFA was removed and washed three times for 10 min with PBS+ (Thermo Fisher Scientific) containing Ca^2+^ and Mg^2+^. Tissues were permeabilized with PBS+ containing 0.3% Triton X‐100 (Sigma–Aldrich) for 30 min on a rocking platform, and then blocked with a blocking solution of PBS+ containing 10% FCS, 2% BSA, and 0.1% Tween for 3 h. Primary antibodies Cardiac Troponin T (1:1500; Abcam), vimentin (1:1000; R&D Systems), and human CD31 (1:200; R&D Systems) were diluted in blocking solution on ice, added to the tissues, and incubated overnight at 4 °C on a rocking platform. The next day, the EHTs were washed three times with PBS+ containing 4% FCS for 20 min on a rocking platform. Secondary antibodies (1:200) were diluted in blocking solution on ice, added to the tissues, and incubated overnight in the dark, at 4 °C on a rocking platform. EHTs were washed three times for 20 min with PBS + containing 4% FCS in the dark and on a shaker. Finally, the tissues were stained with DAPI (1:500) for 1 h at room temperature, in the dark, on a rocker. The tissues on PDMS platforms were transferred to a glass coverslip and placed in the middle of a 500 µm‐thick iSpacer double‐sided sticker with four circular openings (Sunjinlab). Additional PBS+ was added to the wells of the spacer and the samples were sealed with the second glass coverslip. Stained tissues were imaged using a Leica SP8 microscope connected to an Andor Dragonfly 200 spinning disc confocal system, with 20x magnification water immersion objective and Z stack acquisition.

### Statistical Analysis

Each experiment was performed with N ≥ 3, and minimum *n* = 3 tissue recordings per experiment. All graphs show mean ± SEM. Statistical data analysis was conducted in Python using a one‐way ANOVA with a post‐hoc Tukey test. *p*‐values smaller than 0.05 were considered significant differences and denoted with asterisks (*p < 5∙ 10^−2^, **p < 5∙ 10^−3^, ***p < 5∙ 10^−4^, ****p < 5∙ 10^−5^). Statistical significance in graphs was indicated by an asterisk only for p values lower than 0.05. The rest of the values were not considered significant. In the comparison of the three pillar geometries with the same stiffness, the statistical significance was calculated only on day 7 due to the paucity of data points on days 10 and 14 in the case of 90° pillars.

## Conflict of Interest

The authors declare no conflict of interest.

## Supporting information



Supplemental Figure 1

Supplemental Figure 2

Supplemental Figure 3

Supplemental Data

Supplemental Video 1

Supplemental Video 2

Supplemental Video 3

Supplemental Video 4

Supplemental Video 5

Supplemental Video 6

Supplemental Video 7

Supplemental Video 8

## Data Availability

The data that support the findings of this study are available in the supplementary material of this article.
