# Supplementary figures and images for: Tapered Pillar Design for High‐Precision Force Readout in Miniaturized Engineered Heart Tissues From Human Pluripotent Stem Cells
Source: Adv Healthc Mater. 2025 Aug 30;14(31):e01664. doi: 10.1002/adhm.202501664 (PMC12683227; doi:10.1002/adhm.202501664)

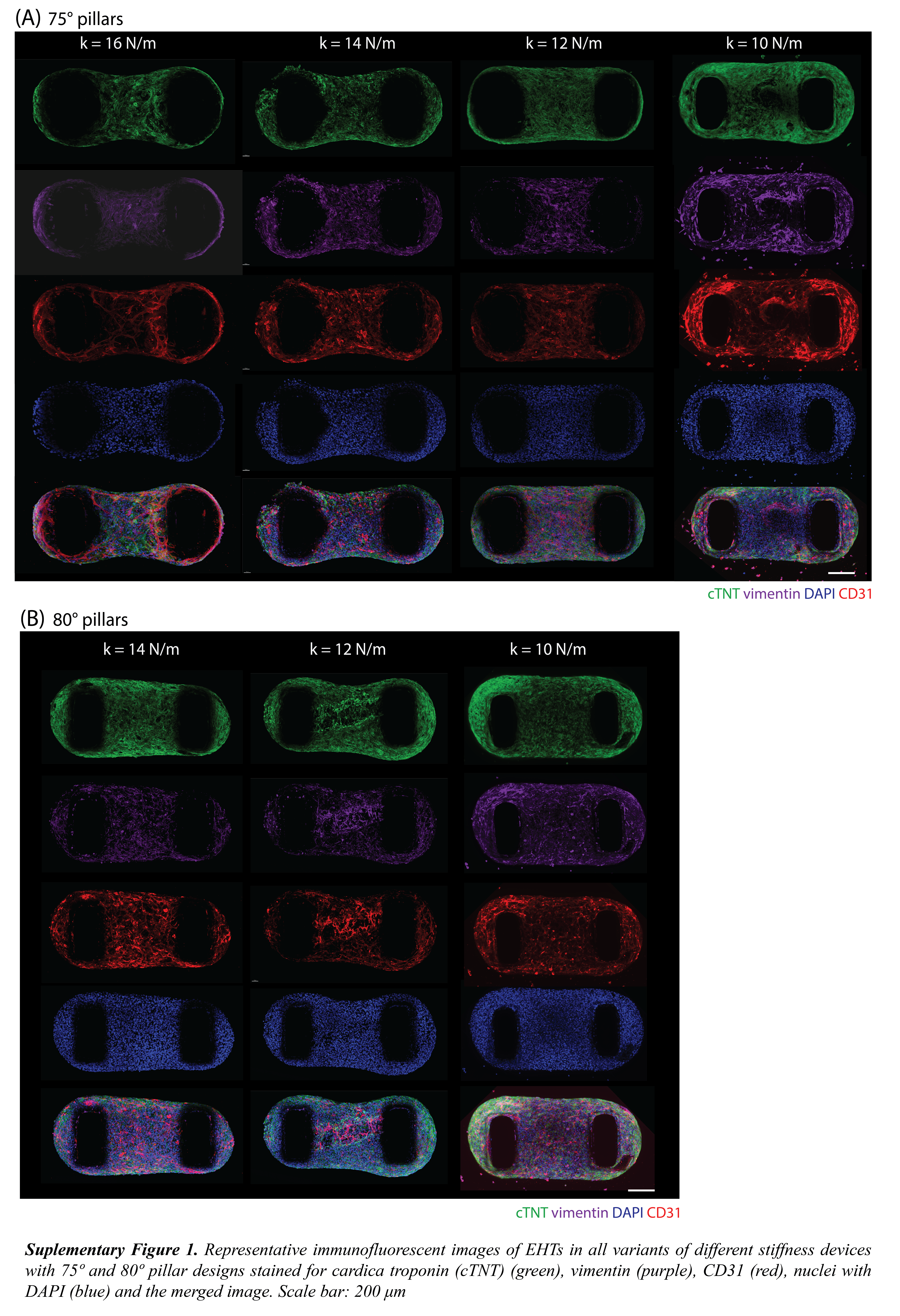

Supplement: Supplementary file 1 — Supplemental Figure 1 [file ADHM-14-0-s011.png]

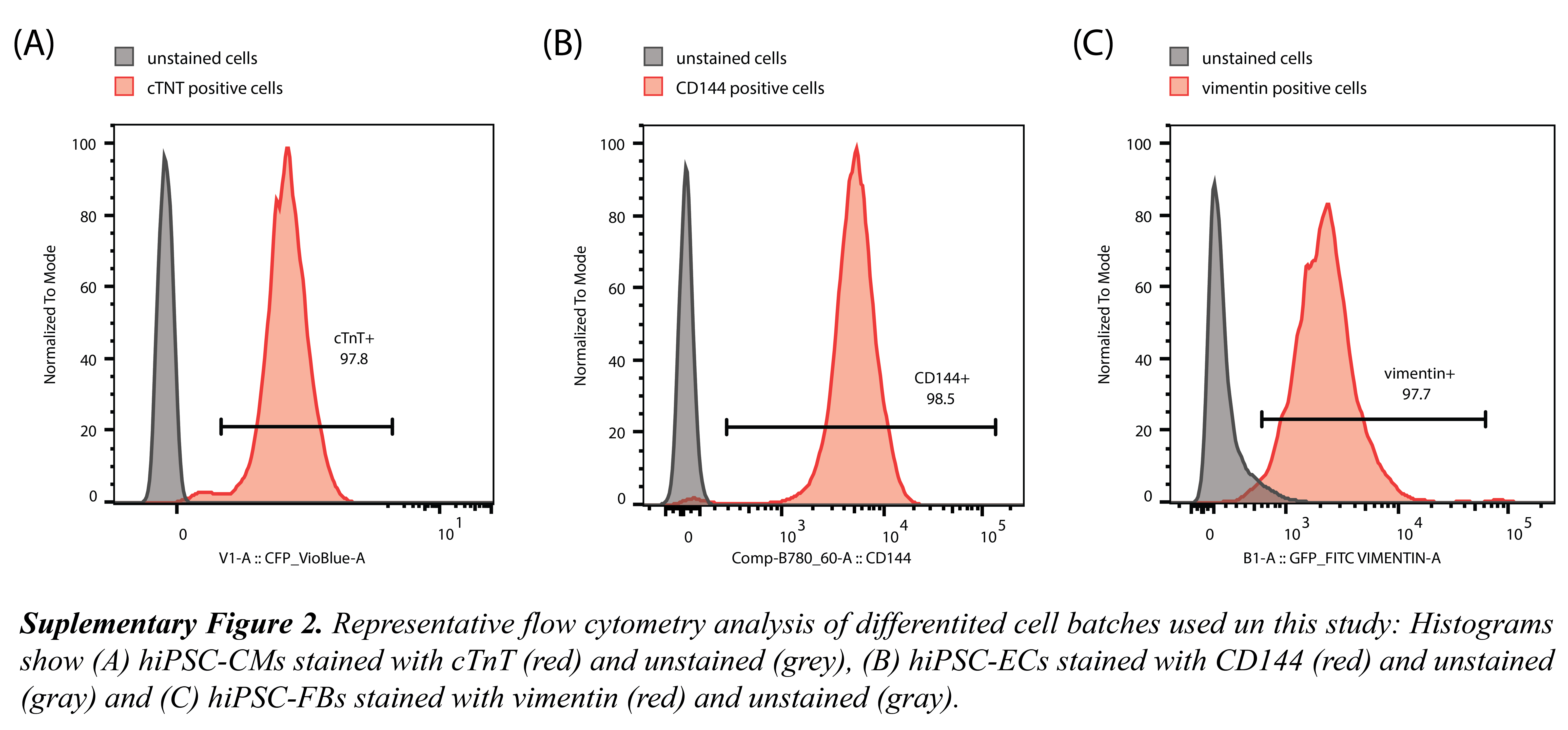

Supplement: Supplementary file 2 — Supplemental Figure 2 [file ADHM-14-0-s004.png]

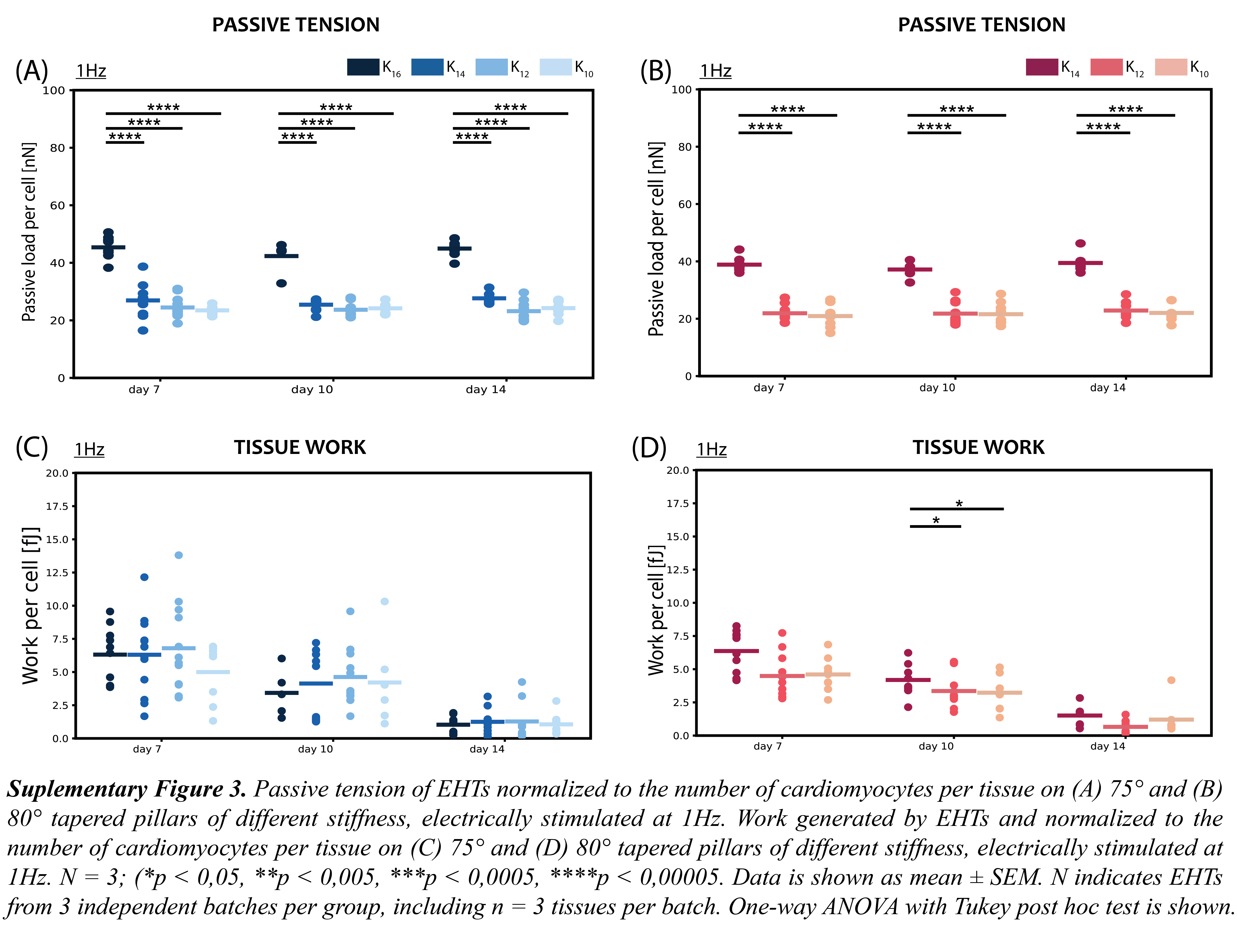

Supplement: Supplementary file 3 — Supplemental Figure 3 [file ADHM-14-0-s008.png]
